# The Prevalence of *Demodex folliculorum* and *Demodex brevis* in Cylindrical Dandruff Patients

**DOI:** 10.1155/2019/8949683

**Published:** 2019-04-03

**Authors:** Jing Zhong, Yiwei Tan, Saiqun Li, Lulu Peng, Bowen Wang, Yuqing Deng, Jin Yuan

**Affiliations:** State Key Laboratory of Ophthalmology, Zhongshan Ophthalmic Center, Sun Yat-Sen University, Guangzhou 510064, China

## Abstract

**Purpose:**

To compare the prevalence of and factors associated with *Demodex brevis* and *Demodex folliculorum* in patients with cylindrical dandruff (CD group) and healthy controls.

**Methods:**

Eyelashes were taken from 1680 patients with CD and 1700 healthy controls in China from March 2015 to May 2017. All patients underwent a complete eye examination, and *Demodex* spp. were counted. The prevalence was analyzed according to age, gender, and clinical features.

**Results:**

Mean patient age was 42.93 ± 16.52 (3–88) and 39.4 ± 13.6 (7–81) years old in the CD and healthy control groups, respectively. In the CD and healthy groups, the positive rate for *Demodex folliculorum* was 27.92% and 8.47%, respectively, while that for *Demodex brevis* was 31.67% and 6.65%, respectively. In the CD group, the prevalence of *Demodex brevis* was higher than that of *Demodex folliculorum*, no matter in the females (33.65% versus 29.01%) or the males (28.54% versus 23.88%) in the CD group. Moreover, the numbers of *Demodex folliculorum* and *Demodex brevis* were significantly and positively correlated with age, in both children and old patients (both *P* < 0.001), as well as with the severity of eyelid congestion (all *P* < 0.05).

**Conclusions:**

In a large sample population, the prevalence of *Demodex brevis* and *Demodex folliculorum* was higher in the CD group than in healthy volunteers. In addition, the severity of eyelid congestion might be exacerbated by the number of *Demodex* spp., which may therefore provide a good clinical reference and objective guide.

## 1. Introduction

*Demodex*, one of the most common parasites in humans, resides in sites with numerous hair follicles and pilosebaceous glands, such as the eyelids [1], face [2], scalp [3], and upper chest [4]. Among more than 140 species of mites, only *Demodex folliculorum* and *Demodex brevis* are found on the human body. *Demodex folliculorum* is approximately 0.3–0.4 mm long, while *Demodex brevis* is approximately 0.2–0.3 mm long [5]. Their life cycle is approximately 14–16 days long, they move mostly at night, and they live in regions such as the sebaceous glands in facial skin, including the nose, nasolabial folds, eyelids, cheek, forehead, chin, and neck [6].

In ophthalmology, ocular demodicosis is typically accompanied by eyelash loss or abnormal alignment and chronic inflammation of the meibomian gland [7], leading to lipid tear deficiency in the conjunctiva [8]; in turn, this deficiency leads to conjunctivitis and sight-threatening keratitis in the cornea [9]. Several studies have also linked the presence of *Demodex* with chronic blepharitis because the mite can perpetuate the follicular inflammatory process [1, 10, 11]. Some researchers consider the mites to be merely passengers on skin because they are found on almost all normal adult skin and thus are coincidentally found on diseased skin [12, 13]. However, clinical observations have revealed that after ineffective conventional therapy, acaricidal therapy can eliminate the clinical symptoms of blepharitis [14]. Nevertheless, direct, absolute proof of a causal relationship has not yet been established because *Demodex* is a host-specific obligate parasite that currently cannot be cultured *in vitro* to parasitize and infect other animal hosts [15]. Therefore, clinical observations based on large samples are important for exploring the relationship between *Demodex* and clinical signs.

Cylindrical dandruff (CD) in the eyelashes, also known as cylindrical casts, are scales that form clear cuffs that collar the lash root and may be composed of keratins and lipids [16, 17]. CD is one of the clinical manifestations of ocular demodicosis, and Tseng's study showed that eyelashes with CD did indeed have a significantly higher rate of *Demodex* infestation than was found in eyelashes without CD [6]. CD in the eyelashes is a common finding in some patients with ocular demodicosis, but whether it is pathognomonic of *Demodex* infestation remains controversial. This debate is partially attributed to the accuracy of methods used to sample and count *Demodex* [18]. Therefore, a modified sampling and counting method was established to enhance the accuracy of *Demodex* diagnosis [6].

However, the exact prevalence of *Demodex* and the pathogenic potential of these mites in eyes with CD remain uncertain. Thus, we performed a study of 1680 patients with CD and 1700 healthy volunteers in China that was designed to determine the prevalence of *Demodex* and the effect of the hosts' factors such as gender, age, and eyelid inflammation score, on the presence or absence of *Demodex*.

## 2. Materials and Methods

### 2.1. Patient Data

**Figure 1:**
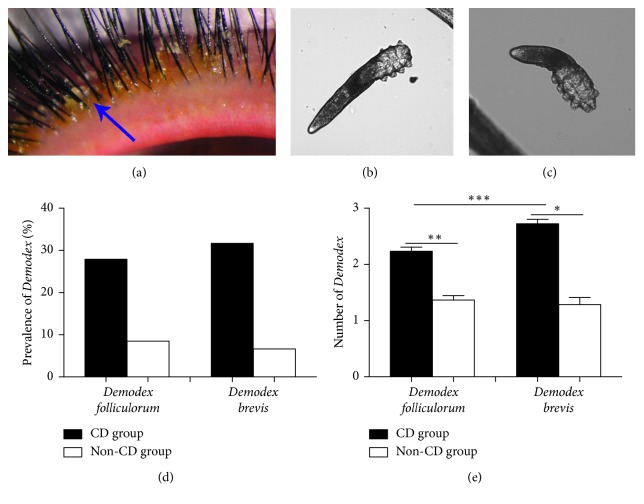
Representative images of cylindrical dandruff (a) (blue arrow; magnification 40x), *Demodex folliculorum* (b), and *Demodex brevis* (c); the prevalence of *Demodex brevis* was higher than that of *Demodex folliculorum*, and the average number of *Demodex folliculorum* was significantly lower than that of *Demodex brevis* (d, e). ^*∗*^*P* < 0.05; ^*∗∗*^*P* < 0.01; ^*∗∗∗*^*P* < 0.001.

A total of 1680 patients with eyelashes showing CD (representative pictures are shown in Figure 1(a)) and who complained of ocular surface irritation and 1700 healthy volunteers who visited our hospital between March 2015 and June 2017 were included in our study. In the healthy group, there were 1166 (68.6%) females and 534 (31.4%) males with a mean age of 39.4 ± 13.6 (7–81) years; in the CD group, there were 1165 (69.4%) females and 515 (30.6%) males with a mean age of 42.9 ± 16.5 (3–88) years. The collected data included basic information such as gender and age, the status of eyelid inflammation, and the results of *Demodex* counting. This study followed the tenets of the Declaration of Helsinki and was approved by the Ethics Committee of the Zhongshan Ophthalmic Center (Guangzhou, China). A total of 3380 individuals in both groups all signed a consent document to participate in the study.

### 2.2. *Demodex* Sampling and Counting

The methods used here were previously described by Kheirkhah et al. [19]. Briefly, two lashes with CD were removed from each lid of each subject by fine forceps and were placed separately on each end of a glass slide for examination under a slit-lamp biomicroscope (SL220; Carl Zeiss, Oberkochen, Germany) at a magnification of ×25. Thus, for each subject, a total of 8 lashes were prepared on 4 slides. A coverslip was mounted on each lash before 20 *μ*L of saline was slowly pipetted at the edge of the coverslip until it surrounded the lash. Then, 20 *μ*L of 100% alcohol (Sigma-Aldrich, St. Louis, MO) was pipetted at the edge of the coverslip; this prolonged the counting time for up to 20 minutes and allowed the embedded *Demodex* to migrate from the CD. Under the microscope, the number of *Demodex* was counted three times, and all samples were photographed in a conventional manner by the same specialist (Doc Tan). The presence of *Demodex* in at least one of the 8 eyelashes was defined as *Demodex*-positive.

### 2.3. Eyelid Inflammation Evaluation

The status of eyelid inflammation was based on the presence of vascular congestion in the eyelid margin, as observed by external photography. These findings were subjectively rated on a four-point scale, as follows: 0, no vascular congestion; 1, mild vascular congestion; 2, moderate vascular congestion; and 3, severe vascular congestion [20].

### 2.4. Statistical Analysis

Data were evaluated using SPSS for Windows 11.5. An unpaired, two-tailed Student's *t*-test was used to compare the numbers of *Demodex brevis* and *Demodex folliculorum* and the numbers of *Demodex* among the different grades of eyelid congestion. The chi-square test and Fisher's exact test were used to evaluate differences in *Demodex* prevalence among different ages and genders. Correlation analysis was used to evaluate the relationship between *Demodex* numbers and age and between *Demodex* rates and eyelid congestion severity. The data were considered significant at *P* < 0.05.

## 3. Results

### 3.1. The Prevalence of Demodex Brevis and Demodex Folliculorum Was Higher in the CD Group than in the Healthy Group

Figures 1(b) and 1(c) show representative microscopic images of *Demodex folliculorum* and *Demodex brevis*. The positive rate of *Demodex folliculorum* was 27.92% and 8.47%, respectively, in the CD group and healthy group, and *Demodex brevis*'s prevalence was 31.67% and 6.65%, respectively, in these two groups (Figure 1(d)). Furthermore, the average number of *Demodex folliculorum* and *Demodex brevis* was 0.52 (0–18) and 0.86 (0–18) in the CD group, which was 0.06 (0–2) and 0.14 (0–2) in the healthy group; the average *Demodex.*spp. number of all the positive subjects was more in the CD group than in the healthy group, no matter in *Demodex folliculorum* (2.23 ± 0.07 versus 1.37 ± 0. 08, *P* < 0.01) or in *Demodex brevis* (2.72 ± 0.07 versus 1.29 ± 0.13, *P* < 0.05). Moreover, the average number of *Demodex brevis* was obviously greater than *Demodex folliculorum* in the CD group (*P* < 0.001) while not in the healthy group (Figure 1(e)). Thus, the prevalence of *Demodex folliculorum* and *Demodex brevis* was higher in CD group compared with that in the healthy group, and the positive rate of *Demodex brevis* was greater than that of *Demodex folliculorum* in the CD group.

### 3.2. The Number of Demodex Brevis Was Higher in Females than in Males

**Figure 2:**
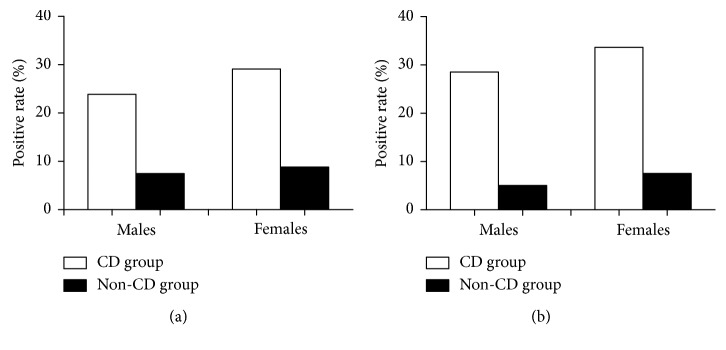
The positive rate of *Demodex folliculorum* (a) and *Demodex brevis* (b) in males and females in the CD and healthy groups. ^*∗*^*P* < 0.05; ^*∗∗*^*P* < 0.01; ^*∗∗∗*^*P* < 0.001.

In the CD group, the positive rate of *Demodex folliculorum* was 23.88% and 29.10%, respectively, in males and females, while in the healthy group, it was 7.49% and 8.83%, respectively, in males and females. The prevalence of *Demodex brevis* showed a trend similar to that of *Demodex folliculorum,* with the positive rate of 28.54% and 33.65% in males and females in the CD group and 5.05% and 7.55% in males and females in the healthy group. The prevalence of *Demodex folliculorum* and *Demodex brevis* were higher in the CD group than in the healthy group in both males and females and higher in females than in males in both groups (Figure 2).

### 3.3. The Number of Demodex Increased with Age in Eyelashes with CD

**Figure 3:**
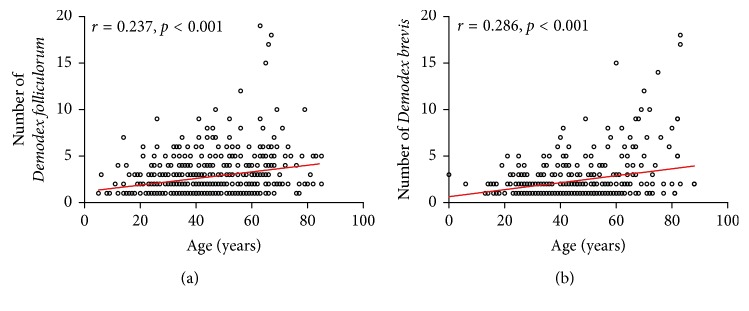
The average number of *Demodex folliculorum* (a) and *Demodex brevis* (b) are significantly correlated with increasing age, from children to older patients. ^*∗*^*P* < 0.05; ^*∗∗*^*P* < 0.01; ^*∗∗∗*^*P* < 0.001.

In the CD and healthy groups, the prevalence of *Demodex folliculorum* was 13.33% and 0% in children (<6 years old), 22.22% and 1.78% in juveniles (7–17 years old), 21.74% and 8.71% in youth (18–40 years old), 24.31% and 9.18% in middle-aged patients (41–65 years old), and 30.97% and 12.87% in old patients (66–88 years old) (Table 1). In the CD and healthy groups, the prevalence of *Demodex brevis* were 26.67% and 2.63% in children, 30.00% and 3.55% in juveniles, 29.55% and 6.46% in youth, 32.67% and 7.75% in middle-aged patients, and 37.42% and 8.77% in old patients. The prevalence of *Demodex brevis* and *Demodex folliculorum* appeared lower in younger age groups than in older age groups, respectively (Table 1). The prevalence differed among different groups, and old patients had the higher prevalence in *Demodex folliculorum* and *Demodex brevis*. Furthermore, in the CD group, the number of *Demodex folliculorum* and *Demodex brevis* per patient was positively correlated with age across all age groups (both *P* < 0.001). The following equations were used: number of *Demodex folliculorum* = 1.180 + 0.035 (age) (*r* = 0.237, *P* < 0.001) (Figure 3(a)) and number of *Demodex brevis* = 0.650 + 0.037 (age) (*r* = 0.286, *P* < 0.001) (Figure 3(b)).

### 3.4. The Prevalence and Number of Demodex Brevis Were Positively Correlated with the Severity of Eyelid Congestion

**Figure 4:**
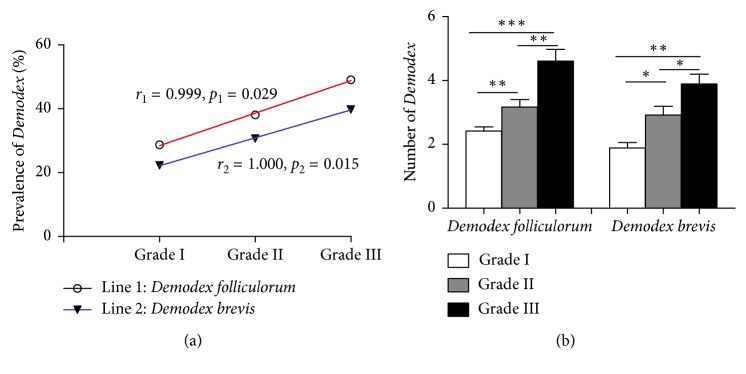
(a) The relationship between congestion severity and the number of *Demodex* in the CD group. (b) In the CD group, the number of *Demodex folliculorum* and *Demodex brevis* was highest in Grade III cases and the lowest in Grade I cases. ^*∗*^*P* < 0.05; ^*∗∗*^*P* < 0.01; ^*∗∗∗*^*P* < 0.001.

In the CD group, we concluded that the severity of eyelid congestion was positively correlated with the prevalence of both *Demodex folliculorum* and *Demodex brevis* (both *P* < 0.05) (Figure 4(a)) according to the following equations: prevalence of *Demodex folliculorum* (%) = 18.25 + 10.19 (grade) (*r* = 0.999, *P*=0.029) and prevalence of *Demodex brevis* (%) = 13.40 + 8.75 (grade) (*r* = 1.000, *P*=0.015). The prevalence of *Demodex folliculorum* increased from 22.26% in Grade I to 30.66% in Grade II to 39.75% in Grade III, while the prevalence of *Demodex brevis* increased from 28.70% to 38.09% and 49.01% in Grade I, II, to Grade III, respectively. Furthermore, the highest numbers of *Demodex folliculorum* and *Demodex brevis* individuals were observed in Grade III cases, whereas the fewest were observed in the Grade I cases (all *P* < 0.05) (Figure 4(b)). Specifically, the prevalence and number of *Demodex folliculorum* and *Demodex brevis* increased with the severity of eyelid congestion.

## 4. Discussion

*Demodex* is a parasite commonly observed on human skin [21], and some investigators have suggested that there is a symbiotic relationship between mites and humans that may even beneficial for the hosts because these mites ingest bacteria that can grow in the follicular canal [22, 23]. However, a growing body of evidence indicates that these mites may also act as pathogens in a number of skin diseases, such as rosacea [24], alopecia [25], and chronic blepharitis [11].

The prevalence of *Demodex folliculorum* and *Demodex brevis* was clearly higher in the CD group than in healthy volunteers in our study; although the positive rate of 27.92% and 31.67% was lower than the prevalence of 100% in Tseng's study [6], it also provided strong evidence to support the high prevalence in CD lashes. The eye is surrounded by protruding body parts such as the nose, brow, and cheek; the eyelid is not as accessible as the face is to daily cleansing hygiene. Therefore, once a *Demodex* infestation is established in the face, it is likely to spread and flourish in the eyelids. Microabrasions caused by the mite's claws can induce epithelial hyperplasia and reactive hyperkeratinization around the base of the lashes, forming CD [26], which is closely associated with *Demodex* infestation. In addition, differences in sample size and regions among studies have led to a lack of consistent results until now. For example, Wesolowska et al. [27] reported that the overall prevalence of *Demodex* spp. is 41% in Poland, a rate of positivity of 37.3% was reported for *Demodex* spp. in Turkish volunteers [28], and a prevalence rate of 21.2% was found in Shangqiu City of Henan Province [29], 36.3% in Tangshan [30], and 51.5% in inner Mongolia [31]. Thus, the difference in prevalence between our and Tseng's results might be normal.

Moreover, we found that the prevalence of *Demodex folliculorum* and *Demodex brevis* was higher in females than in males. The prevalence of *Demodex brevis* was 33.65%, which is similar to the rate of 39.3% found in women in the Malatya province in Turkey [32] but lower than the prevalence of 100% reported in Tseng's study [6]. However, the gender distribution of *Demodex* spp. in the present study was not in agreement with the results of Elston's study [33], in which men were typically more heavily infested than women with *Demodex*. The application of exogenous lipids in cosmetics may also affect the growth of *Demodex* mites in females because females have lower androgen levels, and the meibomian gland is an androgen target organ. Therefore, females may be more susceptible to meibomian gland dysfunction, the resultant lipid insufficiency, and therefore *Demodex* spp. attack [34].

*Demodex* spp. are acquired shortly after birth during nursing and become more abundant during puberty [33]. In our study, the total number of *Demodex folliculorum* and *Demodex brevis* per patient was significantly correlated with increasing age from children to older patients, and their prevalence was significantly higher in older patients than in youths or children. Why do mites proliferate much more in older patients? Some of the physical barrier characteristics of an elderly person's facial skin, such as increased skin pH [35], reduced skin surface hydration levels [36], and abnormal fatty acid composition [37], are conducive to mite proliferation. Additionally, in healthy skin, *Demodex* mites can cause host damage, so they may seize the opportunity to proliferate as immunity decreases or the host becomes immunocompromised [22]. Thus, the elderly, who have comparatively poor sanitary conditions and practices, abnormal skin barriers, and relatively compromised immunity, would be easily invaded by *Demodex* spp. Moreover, the prevalence of *Demodex brevis* was more common than *Demodex folliculorum* in the CD group and healthy subjects, which might be due to the fact that *Demodex folliculorum* resides in the lash follicle, whereas *Demodex brevis* burrows deep into the lash's sebaceous gland and the meibomian gland [38]. Although some studies reported that *Demodex folliculorum* can be more easily isolated than *Demodex brevis* and thus the prevalence of *Demodex folliculorum* was higher compared with the *Demodex brevis* [39], we deduced that the tendency might be different in *Demodex brevis*-related or *Demodex folliculorum*-related ocular diseases, and *Demodex brevis* might be more common in the sebaceous gland- or meibomian gland-related diseases, such as Chalaza [6], while *Demodex folliculorum* was more commonly seen in lash follicle-related diseases, such as posterior blepharitis, or keratoconjunctivitis [38].

In addition to CD, eyelid margin inflammation is one of the main clinical manifestations of ocular demodicosis; thus, the severity of eyelid inflammation may indicate the prognosis [40]. The increased number and extrafollicular localization of mites enhance the probability of a hypersensitivity reaction, inflammation, and the secretion of inflammatory cytokines. Regardless of the prevalence or number of *Demodex folliculorum* and *Demodex brevis*, both were positively correlated with eyelid congestion severity; these results demonstrate that the *Demodex* spp. infestation may act as a pathogen in ocular pathologic features. This result is in agreement with Tseng's results [41].

In conclusion, we explored a large sample population and found that the prevalence of *Demodex brevis* and *Demodex folliculorum* were higher in the CD group than in healthy volunteers. Our results demonstrate that in eyelashes with CD, the prevalence of *Demodex brevis* is higher than that of *Demodex folliculorum*. We also found that the number of *Demodex* spp. increases with age and that females are attacked more easily than males by *Demodex* spp. In patients with CD eyelashes, the severity of eyelid congestion was exacerbated by the prevalence and number of *Demodex* spp. Further studies should focus on the specific mechanism of *Demodex* spp. infection, build diagnostic criteria for eyelid demodicosis, and explore the relationship between *Demodex* spp. and ocular immunology to develop therapies against *Demodex*.

## Figures and Tables

**Table 1:** Distribution of *Demodex folliculorum* and *Demodex brevis* by age in the CD and healthy groups.

Age (years)	*Demodex folliculorum*, positive/*n* (%)		*Demodex brevis*, positive/*n* (%)	
	CD group	Healthy group	CD group	Healthy group
0–6	2/15 (13.33%)	0/38 (0%)	4/15 (26.67%)	1/38 (2.63%)
7–17	20/90 (22.22%)	3/169 (1.78%)	27/90 (30.00%)	6/169 (3.55%)
18–40	142/653 (21.74%)	62/712 (8.71%)	193/653 (29.55%)	46/712 (6.46%)
41–65	186/765 (24.31%)	56/610 (9.18%)	250/765 (32.67%)	46/610 (7.75%)
66–88	48/155 (30.97%)	22/171 (12.87%)	58/155 (37.42%)	15/171 (8.77%)

## Data Availability

The datasets will be provided via a link if required after publication.
